# Role of efavirenz plasma concentrations on long-term HIV suppression and immune restoration in HIV-infected children

**DOI:** 10.1371/journal.pone.0216868

**Published:** 2019-05-16

**Authors:** Nontiya Homkham, Tim R. Cressey, Naim Bouazza, Lily Ingsrisawang, Pornchai Techakunakorn, Jutarat Mekmullica, Thitiporn Borkird, Achara Puangsombat, Sathaporn Na-Rajsima, Jean Marc Treluyer, Saik Urien, Gonzague Jourdain

**Affiliations:** 1 Institut de recherche pour le développement (IRD UMI 174), Marseille, France; 2 Ecole Doctorale de Santé Publique, Université Paris Saclay, Paris, France; 3 Department of Statistics, Faculty of Science, Kasetsart University, Bangkok, Thailand; 4 Faculty of Public Health, Thammasat University, Bangkok, Thailand; 5 Faculty of Associated Medical Sciences, Chiang Mai University, Chiang Mai, Thailand; 6 Harvard T.H. Chan School of Public Health, Boston, Massachusetts, United States of America; 7 Department of Molecular & Clinical Pharmacology, University of Liverpool, Liverpool, United Kingdom; 8 Unité de Recherche Clinique Paris Centre, Assistance Publique-Hôpitaux de Paris, Paris, France; 9 CIC1419, INSERM & APHP, EAU08 Université Paris Descartes Sorbonne Paris Cité, Paris, France; 10 Phayao Provincial Hospital, Phayao, Thailand; 11 Bhumibol Adulyadej Hospital, Bangkok, Thailand; 12 Hat Yai Hospital, Songkla, Thailand; 13 Samutprakarn Hospital, Samutprakarn, Thailand; 14 Mahasarakam Hospital, Mahasarakam, Thailand; Seconda Universita degli Studi di Napoli, ITALY

## Abstract

**Background:**

To access the long term relationship between efavirenz plasma concentrations and evolution of HIV RNA loads and CD4 cell counts in children.

**Methods:**

Retrospective analysis of data from HIV-infected children on first line efavirenz-containing regimen. A population pharmacokinetic-pharmacodynamic (PK-PD) model was developed to describe the evolution of HIV RNA load and CD4 cell count (efficacy outcomes) in relation to efavirenz plasma concentration. Individual CYP2B6 516 G>T genotype data were not available for this analysis. A score (*IS*_*EFV*_) quantifying the effect of efavirenz concentrations on the long-term HIV replication was calculated from efavirenz concentrations and PD parameters and, a value of *IS*_*EFV*_ below which HIV replication is likely not suppressed was determined. Cox proportional hazards regression models were used to assess the association of the risk of viral replication with *IS*_*EFV*_, and with efavirenz mid-dose concentration(*C*_12_).

**Results:**

At treatment initiation, median (interquartile range, IQR) age was 8 years (5 to 10), body weight 17 kg (14 to 23), HIV RNA load 5.1 log_10_ copies/mL (4.6 to 5.4), and CD4 cell count 71 cells/mm^3^. A model of PK-PD viral dynamics assuming that efavirenz decreases the rate of infected host cells adequately described the relationship of interest. After adjusting for age, baseline HIV RNA load and CD4 cell counts an *IS*_*EFV*_ <85% was significantly associated with a higher risk of viral replication (*p*-value <0.001) while no significant association was observed with *C*_12_ <1.0 mg/L.

**Conclusion:**

The *IS*_*EFV*_ score was a good predictor of viral replication in children on efavirenz-based treatment.

## Introduction

Efavirenz in combination with a dual-nucleoside reverse transcriptase inhibitor (NRTIs) backbone is the preferred first line antiretroviral therapy (ART) for HIV-infected children >3 years of age and weighing >10 kg [1]. The use of efavirenz-based ART has been shown very effective in suppressing viral replication in children and restoring immune function [2–3]. Efavirenz has been shown associated with a lower rate of virological failure compared to nevirapine in children [4–5]. However, a variability in response to efavirenz has been ascribed to differences in pharmacologic, virologic, immunologic, and behavioral characteristics [6]. To achieve target drug exposure, dose adjustment strategies have been designed using therapeutic drug monitoring (TDM), which individualizes drug dosing in order to maintain plasma drug concentrations within a target range (therapeutic window) [7–8].

Among children initiating first line efavirenz-based ART in Africa, a higher percentage of those with a minimum efavirenz plasma concentration >1.1 mg/L had an HIV RNA load decrease greater than 2 log_10_ copies/mL after 3 months compared to children below this threshold [9].

A retrospective study in adult Marzolini *et al*. [10], reported that 50% of patients with efavirenz plasma concentration (below 1.0 mg/L) had viral replication (HIV RNA load >400 copies/mL). Among children, lower efavirenz concentrations were associated with higher risk of viral replication >400 copies/mL [11]. Recent data indicates that the efavirenz plasma concentrations may not provide an efficient tool to accurately predict the risk of viral replication [12–13].

Our aim was to predict with more accuracy than efavirenz concentrations alone the risk of viral replication using model taking into account the relationship between exposure to efavirenz and evolution of HIV-1 RNA load and CD4 cell count.

## Materials and methods

### Patients

This analysis includes data from the prospective Program for HIV Prevention and Treatment (PHPT) Observational Cohort (NCT00433030). We selected all antiretroviral HIV-infected children who started as first line an efavirenz-containing ART in between May 1, 2002 and March 30, 2010, had HIV-1 RNA load and CD4 cell count measurements, and at least one efavirenz plasma concentration measurement available at any time point. Efavirenz was prescribed following US FDA approved weight-based dosing guidelines, as capsules or tablets to be taken once a day in the evening without regard for food. Children demographics (sex, age, body weight and height), antiretroviral treatment, adverse events, and laboratory data, including lipid plasma concentrations, alanine transaminase levels, HIV-1 RNA loads and CD4 cell counts were extracted from the cohort study database. Children receiving drugs known to interact with efavirenz, such as rifampicin, were excluded from the analysis.

The PHPT Cohort Study was approved by the ethical committees at the Thai Ministry of Public Health, local hospital ethics committees, and the Faculty of Associated Medical Sciences, Chiang Mai University, Thailand. Written informed consents were obtained from parents/legal guardians, and assents as appropriate.

### HIV-1 RNA load and CD4 cell count measurements

HIV-1 RNA loads and CD4 cell counts were performed before treatment initiation and every 6 months after starting or switching ART. Plasma HIV-1 RNA loads were measured using the Cobas Amplicor HIV-1 Monitor RNA test version 1.5 (Roche Molecular Systems) from 2002 to 2008 and Abbott Real-Time HIV-1 assay (Abbott Molecular) since 2009 at the PHPT central laboratory in Chiang Mai (lower limits of detection, 50 copies/mL for Roche technique and 40 copies/mL for Abbott technique; Comparison of results showed Pearson's correlation coefficient, *r* = 0.95), with quality assured through the Virology Quality Assurance Proficiency Program (VQA). CD4 cell counts were measured using a flow cytometer at each local hospital laboratory, with quality control from the Center of Excellence for Flow Cytometry, Mahidol University, Bangkok, Thailand.

### Population pharmacokinetic analysis

Efavirenz plasma concentration data were predicted using a previously published population PK model generated from the same study population [11]. Efavirenz plasma concentrations over 24 hours were described using a one-compartment model with delayed absorption via two transit compartments. Children were separated into two groups as either ‘fast’ (95% of children) or ‘slow’ metabolizers using a mixture model for efavirenz clearance (CL/F). Allometric scaling best described the relationship between body weight, apparent oral CL/F and volume of distribution.

HIV-1 RNA load and CD4 cell count data according to treatment duration were fitted using a nonlinear mixed effects modelling software program Monolix (Version 4.1.3, wfn.software.monolix.org). The population means and variances for the PD parameters were estimated by computing the maximum likelihood estimator without any approximation of the model (no linearization) using the stochastic approximation expectation maximization (SAEM) algorithm combined to a Markov Chain Monte Carlo (MCMC) procedure [14] (number of chains = 5 for all estimations). Residual variability (*ε*) was investigated using proportional, constant or combined error models. The between-subject variability (BSV or η) was ascribed to an exponential model. The Bayesian information criterion (BIC) was used to compare different models, i.e. structural models, covariate effect(s) on PD parameter(s). residual variability models, and structure of the variance-covariance matrix for the BSV parameters. Age, body weight, height, body mass index z-score (continuous or categorized), alanine transaminase levels, sex and type of NRTI backbone were individually tested as covariate effects. Categorical covariates (CA), e.g. sex, were modelled according to the equation ${IC}_{50}=\theta_{{IC}_{50}}\times B^{CA}$, where *B*^*CA*^ is the estimated influential factor for the categorical covariate (CA = 0,1). Continuous covariates (CO) were systematically tested via generalized additive modelling versus the basic model. For example, for *IC*_50_, the equation ${IC}_{50}=\theta_{{IC}_{50}}{\left[CO/median(CO)\right]}^{B_{CO}}$ was used, where $\theta_{{IC}_{50}}$ is the typical value of *IC*_50_ for an individual with the median covariate value and *B*_*CO*_ is the estimated influential factor for the continuous covariates. A covariate was retained in the model if it (*i*) was biologically plausible, (*ii*) produced a BIC decrease, and (*iii*) produced a reduction in the variability of the PD parameter, assessed be the associated BSV. Goodness-of-fit were assessed graphically.

The PK-PD model was similar to that reported to describe the relationship between the exposure to three antiretroviral drugs (efavirenz, didanosine and lamivudine) and the evolution of HIV-1 RNA load and CD4 cell count in Africa [15]. Following Ribeiro and Perelson [16], three differential equations described the viral dynamics where efavirenz inhibits the production of infected cells:

dT/dt=λ−(d×T)−[I(t)×β×T×V] (1)

$$dT^{*}/dt=[I(t)\times \beta \times T\times V]-(\delta \times T^{*})$$ (2)

$$dV/dt=(p\times T^{*})-(c\times V)$$ (3)

**Fig 1:**
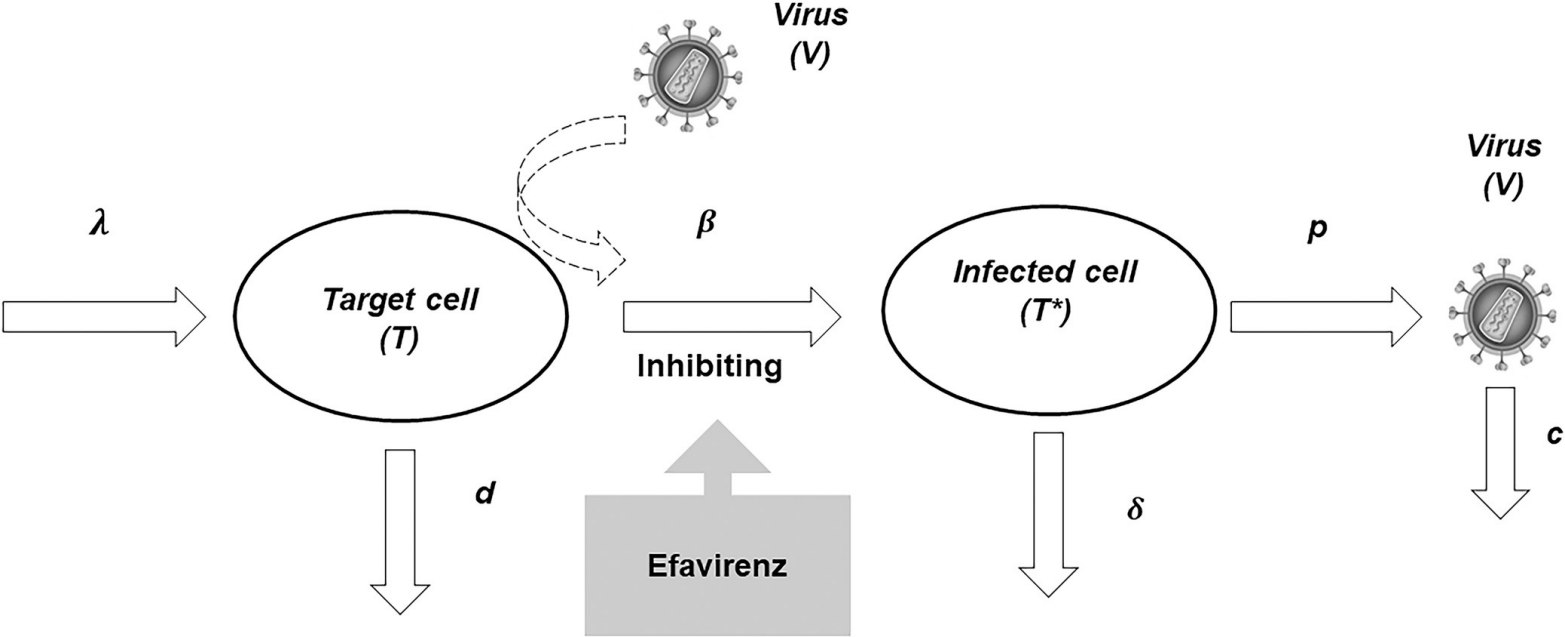
Pharmacokinetic-pharmacodynamic (PK-PD) viral dynamic model in which efavirenz decreases the rate of infected host cells. Abbreviations: *T*; Target cells; T*, infected cells; *V*, free virions; *λ*, production rate constant of uninfected target cells; *d*, elimination rate constant of uninfected target cells; *β*, infected rate constant of target cells; *δ*, elimination rate constant of infected cells; *p*, virion production rate constant; *C*, elimination rate constant of free virions.

These equations represent three compartments including (*i*) uninfected T lymphocyte target cells (*T*), (*ii*) HIV-infected cells (T*) and (*iii*) free virions (*V*). The model assumes that T lymphocyte target cells are produced at a constant rate *λ* and die at rate *d*. Parameters *β* and *δ* are infection and death rates of infected cells. The virus is produced at rate *p* per infected cell and eliminated at rate *c*. Efavirenz, which reduces the ability of HIV virus to infect new target cells by inhibiting the reverse transcriptase enzyme [17], was assumed to decrease the production of infected cells via the inhibition of the parameter *β* (Fig 1). The corresponding inhibition function *I*(*t*) is defined according to the following an E_max_ model [18];

$$I(t)=1-[\left(I_{max}\times C_{av}\right)]/[{IC}_{50}+C_{av}]$$ (4)

where *I*_*max*_ is the maximum fractional inhibition, *IC*_50_ is the efavirenz plasma concentrations producing 50% of the maximal inhibitory effect (mg/L) and *C*_*av*_ is the individual average efavirenz plasma concentration (mg/L). *C*_*av*_ = (*dose*/*dosing interval*)/*CL*_*i*_, where *CL*_*i*_ is the individual efavirenz clearance (L/h). The individual efavirenz clearances for each child were estimated using our previously published population PK model described above [11].

At steady state, before initiating antiretroviral treatment, the initial conditions of the system are *T*_0_ = (*c*×*δ*)/(*β*×*p*), $T_{0}^{*}=\left(c\times {VL}_{0}\right)/p$ and *VL*_0_ = [(*λ*×*p*)/(*c*×*δ*)]−(*d*/*β*) [15].

Initial conditions were then re-formulated in order to estimate HIV-1 RNA load (*VL*_0_) and CD4 cell counts (*CD*_0_) as baseline parameters, allowing the determination of the corresponding between-subject variability. Thus, *λ*, *d*, *β*, *δ*, *p* and *c* were derived from HIV-1 RNA load and CD4 cell count at baseline.

### Model validation

The final population PK-PD model was evaluated using a visual predictive check (VPC). For the VPC, prediction-corrected (Uppsala prediction correction) visual check were used as informative diagnostic tools to allow inspection of model appropriateness across time as well as across covariate values.

### Impact of efavirenz plasma concentrations on viral replication

We defined virological failure as viral replication (HIV-1 RNA load >200 copies/mL) at least after 6 months of treatment and confirmed by a consecutive measurement after 6 months of treatment. The *C*_12_ and *C*_24_ were estimated using our population PK model [11].

From the final model, an inhibitory score (*IS*_*EFV*_) was calculated from *C*_*av*_, the *I*_*max*_, and *IC*_50_ values as follows:

$${IS}_{EFV}=[\left(I_{max}\times C_{av}\right)/\left({IC}_{50}+C_{av}\right)]\times 100$$ (5)

The *IS*_*EFV*_ stands for the resulting effect of efavirenz on HIV replication. A higher *IS*_*EFV*_ indicates a higher effect of efavirenz on HIV replication.

Sex, baseline characteristics (age, body mass index Z-score, alanine transaminase levels, type of NRTI combination, HDL, LDL, total cholesterol and triglycerides), *C*_12_, *C*_24_, *C*_*av*_, *IC*_50_ and *IS*_*EFV*_ during the period of observation were compared between children with or without viral replication using Fisher’s exact test for categorical variables and the Wilcoxon rank-sum test for continuous variables.

A value of the *IS*_*EFV*_ below which HIV replication is likely not to remain suppressed was determined using three methods as Youden’s index, a closest-to-(0,1) criterion and Lui method [19].

The time to confirmed viral replication during the first three years of treatment was estimated by Kaplan-Meier method, and time-to-event distributions were compared using a log-rank test. We estimated the hazard ratio of viral replication between children with *C*_12_ above/below 1.0 mg/L and with *IS*_*EFV*_ above/below cut-off value, after adjusting for potential confounders (HIV-1 RNA load, CD4 cell count and age at treatment initiation) using Cox proportional hazard regression models. The Cox proportional hazards assumption of each model was assessed using Schoenfeld residuals.

## Results

### Study population and follow up

Eighty-seven children (51% boys) met the selection criteria for analysis. Their first-line regimens contained zidovudine (n = 57) or stavudine (n = 30), plus lamivudine (n = 84) or didanosine (n = 3) in combination with efavirenz (dosed according to US FDA approved weight-band dosing guideline). Baseline characteristics are shown in Table 1. The median duration of follow up was 24 months (12 to 34) and a total of 443 HIV-1 RNA load and 447 CD4 cell count measurements were available.

**Table 1:** Baseline characteristics of the 87 HIV-infected children.

Characteristics	Median (Interquartile Range) or Number (%)
Male	44 (51%)
Age (years)	8 (5 to 6)
Bodyweight (kg)	17 kg (14 to 23)
Height (cm)	110 (98 to 124)
Body mass index z-score	0.01 (-1.05 to 1.44)
Normal	58 (67%)
Thinness	24 (27%)
Overweight	5 (6%)
Alanine aminotransferase (IU/L)	30 (19 to 44)
CD4 cell counts (cells/mm^3^)	71 (23 to 262)
HIV RNA load (log_10_ copies/mL)	5.1 (4.6 to 5.4)
Total cholesterol (mg/dL)	133 (106 to 157)
Triglycerides (mg/dL)	135 (85 to 108)
Efavirenz dose (mg/kg)	13.9 (13.0 to 16.2)

Overall, 16 of 87 children (18%) had confirmed viral replication >200 copies/mL during the first 3 years of ART. The median (interquartile rang, IQR) of the first viral replication >200 copies/mL was 3.96 log_10_ copies/mL (2.92 to 4.65). In addition, 24 of 87 children (28%) had confirmed viral replication >50 copies/mL. The median (IQR) of the first viral replication >50 copies/mL was 2.92 log_10_ copies/mL (2.01 to 4.20). The Kaplan–Meier estimate of the cumulative risk of confirmed viral replication >200 copies/mL was 16% (10 to 26) at 12 months of treatment, and 20% (12 to 31) at 36 months. The median time at first viral replication >200 copies/mL was 9.45 months (7.05 to 11.55), and 8.60 months (6.75 to 11.45) for first viral replication >50 copies/mL.

### Population pharmacokinetic-pharmacodynamic analysis

The PK-PD viral dynamic model adequately described the relationship between efavirenz plasma concentrations, viral replication, and evolution of CD4 cell counts over the first 3 years of treatment. All fixed-effect parameters had a residual standard error (RSE%) below 25%, except for the efavirenz *IC*_50_ (RSE = 47%). All random effect parameters had a RSE below 34%. After the maximum inhibitory effect (*I*_*max*_) was fixed to 100% (not identifiable), the estimated mean efavirenz *IC*_50_ was 0.1 mg/L. The infected cells (*δ*) elimination rate constant was fixed to 15.2 per month [20]. Between-subject variability parameters were retained for (*i*) production and elimination rates of uninfected target cells, (*ii*) infected rate of infected cells, (*iii*) production and elimination rates of free virions, and (*iv*) *IC*_50_. The residual variability was best described by an additive error model for HIV-1 RNA load and CD4 cell count data. None of the covariates had a significant effect on PD parameters. The final population PD parameters are summarized in Table 2.

**Table 2:** Parameter estimates for the pharmacokinetic-pharmacodynamic (PK-PD) viral dynamic model.

Parameter	Mean	RSE (%)	Parameter	Mean	RSE (%)
*Fixed effects*			*Between-subject variability*		
*λ*—cells/mm^3^/month	89.2	8	*ω*_*λ*_	0.6	16
*d*—cells/mm^3^/month	0.1	11	*ω*_*d*_	0.6	34
*β* ×10^−6^—per month	6.7	17	*ω*_*β*_	1.0	15
*δ*—per month (fixed)	15.2	-	*ω*_*p*_	0.4	23
*p* ×10^4^—per month	6.5	10	*ω*_*c*_	0.5	14
*c*—per month	3.3	10	$\omega_{{IC}_{50}}$	2.1	19
*I*_*max*_ (fixed)	1.0	-	*ω*_*λ*,*d*_	0.6	21
*IC*_50_—mg/L	0.1	47	*Residual variability*		
			*σ*_*VL*_—log_10_ copies/mL	0.7	4
			*σ*_*CD*4_–cells/mm^3^	176.0	4

Abbreviations. RSE%, relative standard error (standard error of estimate / estimate×100); *λ*, production rate constant of uninfected target cells; *d*, elimination rate constant of uninfected target cells; *β*, infection rate constant of target cells; *δ*, elimination rate constant of infected cells; *p*, production rate constant of free virions; *c*, elimination rate constant of free virions; *I*_*max*_, maximum fractional inhibition of efavirenz on the production of infected cells; *IC*_50_, efavirenz plasma concentration at which effect reaches 50% of its maximum; *ω*, between-subject variability estimates; *σ*, residual variability estimate; *VL*, HIV RNA load; *CD*4; CD4 cell count.

### Model evaluation

The VPCs of the final model, i.e. median, 25^th^ and 75^th^ percentile curves drawn over observed HIV-1 RNA loads and CD4 cell counts as a function of treatment duration, showed that the median, 25^th^ and 75^th^ percentiles of observed values were within the corresponding 90% confidence interval of simulated percentiles (S1 Fig).

### Efavirenz plasma concentrations and viral replication

Regardless of metabolizer phenotypes, the median (IQR) *C*_12_ was 2.9 mg/L (2.0 to 3.7), *C*_24_ 1.1 mg/L (0.7 to 1.4) and *C*_*av*_ 1.7 mg/L (1.3 to 2.2). From Eq 5, the median *IS*_*EFV*_ score calculated was 95% (86 to 96).

Baseline characteristics and estimated *C*_12_, *C*_24_ and *C*_*av*_ were not significantly different between the 16 children with viral replication >200 copies/mL and the 71 virologically suppressed. However, both *IC*_50_ and *IS*_EFV_ significantly differed (*p*-value <0.001) between children with and without viral replication (median *IC*_50_: 0.60 versus 0.07; median *IS*_*EFV*_: 63% versus 96%, respectively).

Overall, 10 of 87 children had *C*_12_ <1.0 mg/L and 77 children (89%) had *C*_12_ ≥1.0 mg/L. Among the 10 children with a *C*_12_ <1.0 mg/L (target threshold for efavirenz efficacy), three (30%) had confirmed viral replication compared with 13 (17%) of those 77 with *C*_12_ above 1.0 mg/L (*p*-value = 0.383). Using the Youden’s index, an 85% *IS*_*EFV*_ cut-off value provided a sensitivity of 94% and a specificity of 89% to predict viral replication, and classification accuracy was 90%. An 50% *IS*_*EFV*_ cut-off value using the closet-to-(0,1) criterion provided a sensitivity of 7%, a specificity of 99% and a classification accuracy of 82%. Using Lui method, an 78% *IS*_*EFV*_ cut-off value provided sensitivity of 63%, a specificity of 92% and a classification accuracy of 86%. The *IS*_*EFV*_ cut-off value of 85% provided a high sensitivity, high specificity and high classification accuracy. Using this *IS*_*EFV*_ cut-off, 15 of 23 children (65%) with a score below cut-off had viral replication compared with 1 of the 64 children (2%) with a score above cut-off (log-rank, *p*-value <0.001).

**Fig 2:**
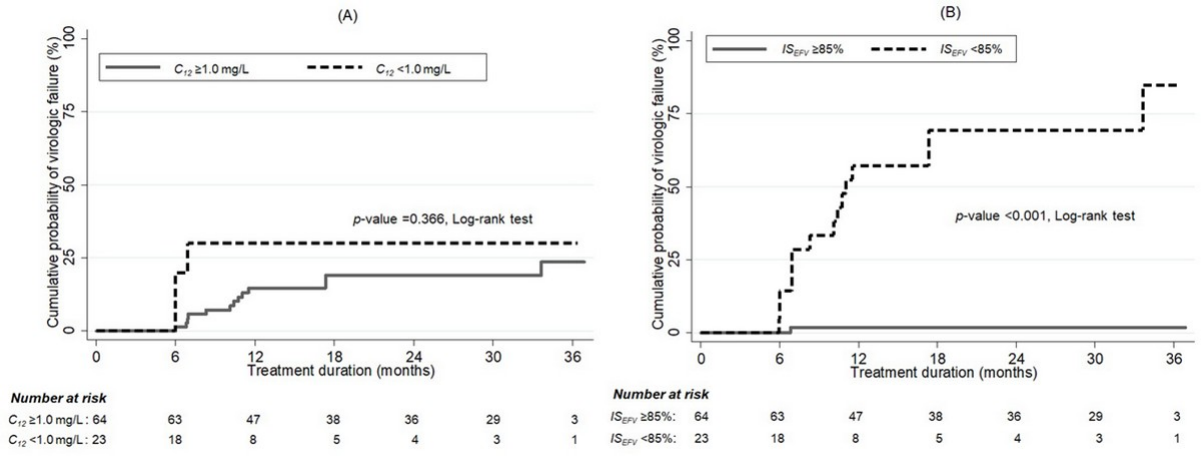
**Cumulative probability of viral replication as a function of treatment duration between *C***_**12**_ **below and above 1.0 mg/L (A), and *IS***_***EFV***_ **below and above 85% (B).** Abbreviations: *C*_12_, efavirenz mid-dose concentration; *IS*_*EFV*_, inhibitory score.

The cumulative risk of viral replication was not different in children with *C*_12_ below and above 1.0 mg/L in the univariate analysis, or adjusting for HIV-1 RNA load, CD4 cell count and age at treatment initiation (adjusted hazard ratio [aHR], 2.74 [95% CI, 0.65 to 11.55], *p*-value = 0.170) (Fig 2A). In contrast, it was significantly higher in children with *IS*_*EFV*_ <85% than children with *IS*_*EFV*_ ≥85% in the univariate analysis or adjusted for HIV-1 RNA load, CD4 cell count and age at treatment initiation (aHR, 67.17 [95% CI, 8.29 to 544.45], *p*-value <0.001) (Fig 2B).

## Discussion

A PK-PD viral dynamics model assuming that efavirenz decreases the rate of infected host cells production adequately described the relationship between efavirenz plasma concentrations and efficacy outcomes (HIV-1 RNA load and CD4 cell count evolutions) over the first 3 years of treatment. However, there was no significant difference in the time to viral replication between children with *C*_12_ below and above 1.0 mg/L. This finding is consistent with previous studies in adults and children starting efavirenz-based regimen. In a study in Cambodian adults, efavirenz plasma concentrations (12 to 16 hours after post-dose) below 1.0 mg/L were not significantly associated with virologic failure (HIV RNA load >250 copies/mL) [21]. This was similar to a study in African children, where there was no significant difference in the risk of virologic failure (HIV RNA load >400 copies/mL) according to efavirenz plasma concentration [22]. While, a study A5202 found an interaction between weight and low efavirenz concentration (at least one plasma concentration <1mg/L) was associated with virologic failure (defined as a confirmed HIV-1 RNA load ≥1,000 copies/mL at or after 16 weeks and before 24 weeks or ≥200 copies/mL at or after 24 weeks) [23]. Similarly, an adult study in South Africa found higher mid-dose efavirenz plasma concentration was associated with decreased risk of virologic failure (HIV RNA load >400 copies/mL at Week 16 and >40 copies/mL at Week 48), but they found a new threshold of mid-dose efavirenz concentration with 0.7 mg/L was the most predictive of virologic failure [24].

In our study, we defined a novel inhibitory score based on a PD hypothesis, *IS*_*EFV*_, calculated from *C*_*av*_, the maximum possible effect of efavirenz on HIV replication (*I*_*max*_) and efavirenz plasma concentrations producing 50% of the maximal effect of drug (*IC*_50_). The *IS*_*EFV*_ accounted for the contribution of individual efavirenz plasma concentration and viral susceptibilities, and appeared to provide a better predictor of viral replication during the first 3 years of treatment than drug plasma concentration or viral susceptibilities alone. The risk of viral replication was significantly higher in children with a *IS*_*EFV*_ below 85%.

Of note, an inhibitory quotient (IQ) was calculated from actual or expected trough drug concentration (*C*_24_) divided by a measure of viral susceptibilities to that drug, *IC*_50_. In adults, a study published in 2003 in patients on lopinavir/ritonavir with or without efavirenz found that baseline efavirenz IQ was correlated with the 24^th^ week virologic response (HIV RNA load below 400 copies/mL) but the logarithmically transformed efavirenz concentration was not significantly correlated with virologic response [25]. Another study in children calculated a composite inhibition score (CIS), based on the same PD hypothesis as in our analysis, using the plasma concentrations of three drugs (efavirenz, didanosine and lamivudine) to estimate the relative potency of each drug, and efavirenz accounted for 65% of the total effect. A low CIS was associated with the risk of viral replication [15].

There are some limitations to our findings. Firstly, our PK-PD model was developed using data from HIV-infected children, median age 8 years, in Thailand, who may differ from populations previously studied in terms of frequencies of gene polymorphisms affecting efavirenz metabolism [26–27]. A study in Thailand found 48%, 44% and 11% of children with CYP2B6 516G/G, G/T, and T/T genotypes, respectively. The CYP2B6 516G>T gene polymorphisms were strongly associated with higher efavirenz plasma concentration (mean ±SD of efavirenz plasma concentrations were 1,604 ±729 ng/mL for children with G/G genotype, 2,635 ±1,199 ng/mL for G/T genotype and 11,582 ±2,972 for T/T genotype; *p*-value <0.001) [28]. Of note, the individual CYP2B6 516G>T genotype results were not available for the population studied but we took into account the presence of a small proportion of ‘slow’ metabolizers in the studied population. Secondly, the risk of viral replication may also be related to the NRTI backbone. However, in our model, there was no significant association with the NRTI backbone combinations. Thirdly, our PK-PD model was developed to investigate the effect of efavirenz on HIV-RNA time-course and CD4 cell count based on data from HIV-infected children initiating efavirenz-based combination in Thailand; thus the data may not describe a population with different antiretroviral combinations, races and/or ages.

In conclusion, the target threshold of efavirenz plasma concentration <1.0 mg/L may not be an optimal marker of the risk of viral replication. The *IS*_*EFV*_ based on a PD hypothesis was a better predictor of confirmed viral replication during the first 3 years of treatment than *C*_12_.

## Supporting information

S1 Fig Visual predictive checks (VPC) for HIV-1 RNA loads (A) and CD4 cell counts (B) after efavirenz initiation. The observed HIV-1 RNA loads and CD4 cell counts are displayed using blue points and the censored data (simulated from the model) using red points. The green lines show the 25th, 50th and 75th percentiles of observed HIV-1 RNA loads and CD4 cell counts. The blue shaded areas represent the 90% CI around the simulated 25^th^ and 75^th^ percentiles, and the pink shaded areas represent the 90% CI around the predicted median. Abbreviations: CI, confidence interval.
(TIF)
