# Supplementary figures and images for: Role of efavirenz plasma concentrations on long-term HIV suppression and immune restoration in HIV-infected children
Source: PLoS One. 2019 May 16;14(5):e0216868. doi: 10.1371/journal.pone.0216868 (PMC6521995; doi:10.1371/journal.pone.0216868)

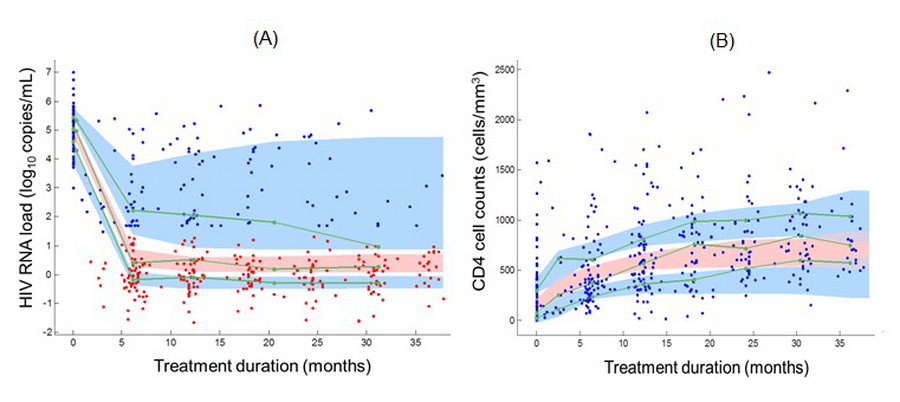

Supplement: S1 Fig — The observed HIV-1 RNA loads and CD4 cell counts are displayed using blue points and the censored data (simulated from the model) using red points. The green lines show the 25th, 50th and 75th percentiles of observed HIV-1 RNA loads and CD4 cell counts. The blue shaded areas represent the 90% CI around the simulated 25th and 75th percentiles, and the pink shaded areas represent the 90% CI around the predicted median. Abbreviations: CI, confidence interval. (TIF) [file pone.0216868.s001.tif]
